# Decreased 5-Hydroxymethylcytosine Is Associated with Neural Progenitor Phenotype in Normal Brain and Shorter Survival in Malignant Glioma

**DOI:** 10.1371/journal.pone.0041036

**Published:** 2012-07-19

**Authors:** Brent A. Orr, Michael C. Haffner, William G. Nelson, Srinivasan Yegnasubramanian, Charles G. Eberhart

**Affiliations:** 1 Department of Pathology, Johns Hopkins University School of Medicine, Baltimore, Maryland, United States of America; 2 Department of Ophthalmology, Johns Hopkins University School of Medicine, Baltimore, Maryland, United States of America; 3 Department of Oncology, Johns Hopkins University School of Medicine, Baltimore, Maryland, United States of America; 4 Sidney Kimmel Comprehensive Cancer Center, Johns Hopkins University, Baltimore, Maryland, United States of America; 5 Brady Urological Institute, Johns Hopkins University, Baltimore, Maryland, United States of America; Geisel School of Medicine at Dartmouth, United States of America

## Abstract

Epigenetic modification of DNA by cytosine methylation to produce 5-methylcytosine (5mC) has become well-recognized as an important epigenetic process in human health and disease. Recently, further modification of 5mC by the ten eleven translocated (TET) family of enzymes to produce 5-hydroxymethylcytosine (5hmC) has been described. In the present study, we used immunohistochemistry to evaluate the distribution of 5hmC in human brain during different periods of development and in a large series of gliomas (n = 225). We found that during development, 5hmC levels are high in more differentiated compartments like the fetal cortex, but low in the periventricular progenitor cell regions. In adults, we found 5hmC levels to be highest in the cortex, but present in all intrinsic cell types in the brain including stromal elements. In brain tumors, 5hmC levels were high in low grade tumors and reduced in malignant glioma, but did not exhibit any correlation with *IDH1* mutation status. Additionally, we identified a significant relationship between low levels of 5hmC and reduced survival in malignant glioma. This observation was further supported by *in silico* analysis showing differential expression of genes involved in 5hmC homeostasis in aggressive subsets of glioblastoma. Finally, we show that several genes involved in regulating the levels of 5hmC are also prognostic in malignant glioma. These findings suggest that 5hmC regulation in malignant glioma may represent an important determinant of tumor differentiation and aggressive behavior, as well as a potential therapeutic target.

## Introduction

Modification of CpG dinucleotides by methylation is an important epigenetic mechanism involved in the regulation of tissue specific gene expression and cellular differentiation [1]. Accumulation of 5mC marks, especially in gene promoter regions, has been shown to be associated with repressed chromatin states and transcriptional silencing [2]. Recent evidence suggests that a group of enzymes of the ten-eleven-translocated family (*TET1-3*) can further convert 5mC to 5-hydroxymethylcytosine (5hmC) in an alpha-ketoglutarate dependent oxidation reaction [3], [4]. 5hmC is detectable in a variety of tissues and can show a differentiation specific distribution pattern [5]–[7]. Although the function of the 5hmC mark is largely unknown, 5hmC could play a role in epigenetic fine tuning and may represent an intermediate in the active demethylation of 5mC [4], [8]–[12].

In the brain, 5hmC has been found to be enriched in purkinje cells of the cerebellum, the cortex, and midbrain of mice [6], [7], [13]. Most studies evaluating the distribution of 5hmC in the brain have relied primarily on biochemical or molecular biological methods that do not allow the evaluation of 5hmC on a cell-by-cell basis [7], [14], [15]. Furthermore, most studies to date have evaluated 5hmC in rodent models and information with respect to the tissue distribution of 5hmC within the adult and fetal human brain is limited [6], [7], [14], [15].

Haffner et al. have recently developed a robust immunohistochemical detection method for 5hmC, which allowed the evaluation of 5hmC in formalin-fixed and paraffin embedded tissue [5]. Using this approach, Haffner et al. reported that 5hmC content was very low in the stem cell/progenitor cell compartments in multiple tissues including intestine, cervix, and skin, but significantly higher in terminally differentiated cells [5]. Furthermore, that study found a significant reduction of global 5hmC content in multiple human cancers compared to their normal counterparts, a finding that has been confirmed by other reports [5], [16], [17] . One recent study reported results from mass spectrometry studies demonstrating a reduction in 5hmC levels in a small series of astrocytomas compared to normal brain [16]. However, the cell-to-cell distribution of 5hmC content in the central nervous system and in brain tumors has not been carefully examined.

A large proportion of low grade gliomas and a smaller fraction of glioblastomas show mutations in the isocitrate dehydrogenase genes (*IDH1* or *IDH2*) [18]–[20]. The normal function of the IDH1/2 proteins is to catalyze the interconversion of isocitrate to alpha-ketoglutarate (α-KG); whereas, the mutant proteins instead produce the metabolite 2-hydroxyglutarate (2-HG) [21]. Although the mechanism is unclear, *IDH1/2* mutations are thought to represent an early event in gliomagenesis and have been associated with a glioma-CpG island methylator phenotype (G-CIMP) and improved prognosis in glioblastoma [18], [22]–[24]. Some recent reports have suggested that production of 2-HG by *IDH1/2* mutations reduce 5hmC levels in tumors by competitively inhibiting the TET enzymes [17], [25], [26]. However, others have failed to detect a relationship between low 5hmC levels and mutant *IDH1/2* in astrocytomas [16], [27].

In this report, we use immunohistochemistry to evaluate the 5hmC content in the human adult brain, the pediatric brain, and the fetal brain during development. Furthermore, we evaluated a panel of 225 human brain tumors for 5hmC. We found that 5hmC content is reduced in high grade tumors compared to low grade tumors and normal brain. Additionally, we identified no relationship between *IDH1* mutation status and 5hmC levels. Finally, we report that reduced 5hmC content is associated with poor prognosis in adult glioblastoma and anaplastic astrocytoma.

## Materials and Methods

### Ethics Statement

Human brain tumor samples and normal control tissue from autopsy specimens were obtained from the archives of the Johns Hopkins Hospital Department of Pathology following appropriate institutional review board approval. No informed consent (verbal or written) was obtained from the retrospective tissue specimens. The research ethics committee waived the requirement for informed consent for samples included in the tissue microarray. The patient data was anonymised prior to use in the study.

### Human Tissues and Tissue Microarrays

Classification of each tumor by subtype was performed according to World Health Organization guidelines [28]. Formalin fixed, paraffin embedded tissue was utilized to construct tissue microarrays according to standard procedures at the Johns Hopkins tissue microarray core facility [29]. Four cores of each tumor were used per array. Tumors containing less than two evaluable cores in the array were excluded from analysis. For 5hmC and 5mC immunohistochemistry, samples were assessed using a semi-quantitative system to construct an H-score, obtained by multiplying the intensity of the stain (0: no staining; 1: weak staining; 2: strong staining) by the percentage (0 to 100) of cells showing that staining intensity (H-score range, 0 to 200). Only nuclear staining in tumor cells was evaluated for 5hmC and 5****mC. For *IDH1* R132H-mutant-specific immunohistochemistry, cytoplasmic reactivity of any intensity within tumor cells was considered positive, whereas the absence of reactivity with the cytoplasm was considered negative.

### Immunohistochemistry

Immunohistochemical detection of mutant *IDH1* was performed using a monoclonal antibody against the *IDH1* R132H mutant (Dianova, Cat# DIA H09 LM, Hamburg, Germany) at a dilution of 1∶50. For detection, the Ultraview universal DAB detection kit was used according to the manufacturer’s instructions (Ventana, Cat# 253–4290, Tucson, Arizona). Immunohistochemical detection of 5hmC and 5mC was performed as described previously [5]. Briefly, paraffin sections were de-paraffinized and rehydrated followed by antigen retrieval consisting of steaming for 30 min in citrate buffer (pH 6.0) followed by incubation in 3.5 N HCl for 15 min at room temperature. For immunolabeling of 5hmC, the rabbit polyclonal 5-hydroxymethylcytosine specific antibody (Active Motif, Cat # 39769, Carlsbad, CA) was applied at 1∶20,000 dilution. For 5mC detection, the mouse monoclonal 5 methylcytosine specific antibody (Calbiochem, EMD Chemicals Inc., San Diego, CA) was used at 1∶2000 dilution. Both primary antibodies were incubated for 1 hour at room temperature. For detection of TET2, slides were steamed in 1 mM EDTA (ph 8.0) for 45 min and incubated with goat-polyclonal TET2 antibodies (Everest Biotech, Cat# EB09642, Oxfordshire, UK) at 1∶300 dilution. Immuno-complexes were detected using the the PowerVision+™ immunohistochemistry detection system from ImmunoVision Technologies Co (Norwell, MA, USA) with 3,3′-diaminobenzidine tetrahydrochloride (DAB) as the chromogen. After immunohistochemical staining, tissue sections were counterstained with hematoxylin.

### Survival and in silico Analysis

Data from The Cancer Genome Atlas (TCGA) was downloaded from the cabio portal (http://www.cbioportal.org/ *)* on February 2, 2012. Z-normalized expression values were used for all gene expression analysis. Survival information was downloaded from the TCGA data portal (http://tcga-data.nci.nih.gov/tcga/). For evaluation of glioblastoma subtypes, the 206 tumors reported by Verhaak et al. were evaluated [30]. For survival analysis, expression and survival data were downloaded for 575 glioblastomas. Samples lacking a complete dataset (expression and survival data) were excluded. Additional expression and clinical data were acquired from the Repository for Molecular Brain Neoplasia Data (REMBRANDT) database (https://caintegrator.nci.nih.gov/rembrant/) on January 18, 2012. A total of 315 glioma samples were evaluated after exclusion of tumors in which the clinical grade was not known. Analysis within tumor grade was performed by evaluating the survival in 181 glioblastomas, 50 grade II astrocytomas, 44 grade III astrocytomas, 19 grade II oligodendrogliomas, and 21 grade III astrocytomas. For 5hmC survival analysis, H-scores were separated into a high 5hmC group and low 5hmC with cutoff point being the first quartile within individual tumor types. For TCGA survival analysis of the TET genes, low expression was designated as the first quartile. For evaluation of the APOBEC genes high level expression was designated as the fourth quartile. For survival analysis of REMBRANDT data, a threshold of 2-fold overexpression was used to designate the high versus low expression group.

### Statistical Analysis

Statistical analyses were performed using the Graph-Pad Prism 4 software unless otherwise specified (GraphPad Software, La Jolla, CA). H-scores derived from 5hmC immunohistochemistry of tissue microarrays were compared between different histologic tumor types or *IDH1* status within tumor type using the unpaired Student’s t-test. For evaluation of gene expression within molecular subtypes of glioblastoma, mean expression was evaluated by Student’s t-test. For survival analysis, the percent survival was calculated using the product limit (Kaplan-Meier) method. Curve comparisons were evaluated using the log-rank test (Mantel-Haenszel). Additional univariate and multivariate survival analyses were performed using the Cox proportional hazards model in the SPSS statistical package (SPSS, Chicago, IL). For all analyses, differences were considered significant at p<0.05.

## Results

### 5hmC Immunohistochemistry in Human Brain at Different Developmental Time Points

**Figure 1:**
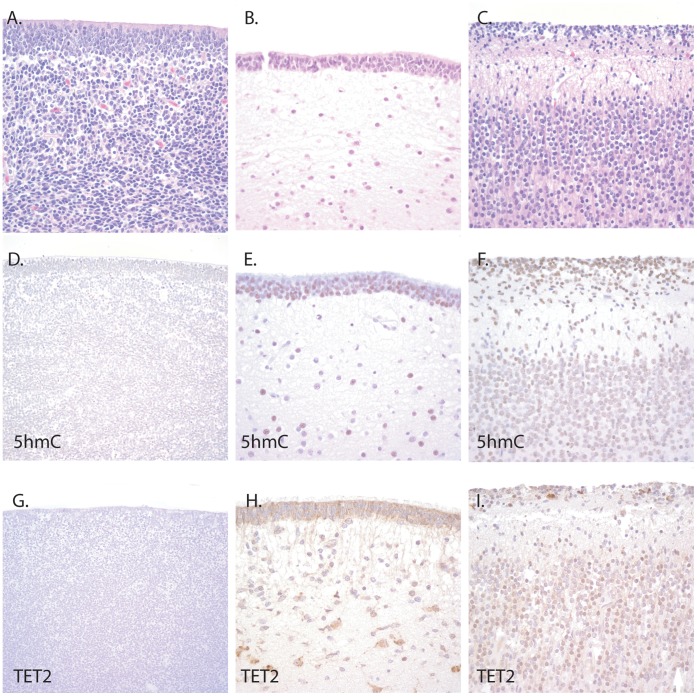
5hmC levels are reduced in the germinal regions of the human fetal forebrain. Fetal brain sections were stained with hematoxylin and eosin (A-C) and evaluated for 5hmC or TET2 levels using immunohistochemistry. Sections of the forebrain germinal region stained with antibodies for 5hmC (D) showed markedly reduced levels. In contrast, periventriclular regions outside the forebrain germinal matrix (E) and in the fetal cortex (F) showed high levels of 5hmC staining. TET2 immunohistochemistry (G-I) showed a similar distribution to 5hmC staining. Original magnification was 200X for all photomicrographs, except panels A, D, and G which were 100X.

To evaluate the tissue distribution of 5hmC we used a chromogenic immunohistochemical staining method described by Haffner *et al* [5]. The antibody as well as the staining method has been shown to be highly specific for 5hmC rather than 5mC by multiple different methods [5], [6], [31], [32]. First, to determine the distribution of 5hmC during different stages of development, we stained autopsy tissues from 3 fetal, 5 pediatric, and 2 adult brains with 5hmC specific antibodies. During development, 5hmC staining was low in the germinal matrix of the fetal forebrain, a region known to harbor a large population of neural progenitor cells (see Figure 1A and D). In contrast, the periventricular regions outside the germinal matrix showed relatively high levels of 5hmC (Figure 1B and E). Additionally, areas containing more differentiated neuronal cells such as the fetal cortex showed high levels of 5hmC staining (Figure 1C and F).

TET enzymes catalyze the production of 5hmC [3], [4]. We therefore searched geodatasets (http://www.ncbi.nlm.nih.gov/sites/GDSbrowser/) for experiments evaluating TET gene expression over the course of neurogenesis. In the one relevant murine dataset identified (GDS3442), *TET2* showed the greatest increase in expression during neurogenesis as compared to the other TET isoforms, *TET1* and *TET3* (Figure S1). We were able to confirm this using immunohistochemistry for TET2 in human fetal brain, with low levels in the progenitor regions of the subventricular zone and high levels in the fetal cortex, nearly identical to the distribution of 5hmC (Figure 1G-I).

**Figure 2:**
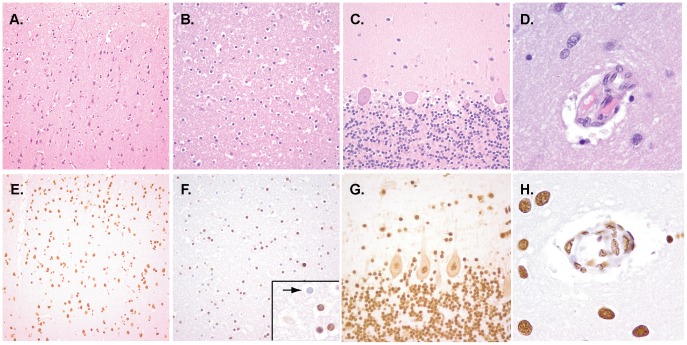
The adult human brain contains relatively high levels of 5hmC. Sections from multiple regions in the pediatric and adult human brain were stained for hematoxylin and eosin (A-D) and by immunohistochemistry for 5hmC (E–F). Both the pediatric and adult brain showed relatively strong staining for 5hmC in grey matter of the cerebral cortex (E), white matter (F), and granule and purkinje cell neurons of the cerebellum (G). The white matter showed some variability, with many 5hmC positive cells admixed with immunonegative cells (see inset of panel F, arrow). Additionally, stromal elements such as the cerebral vasculature (H) also showed relatively high levels of staining. The original magnification for D,H, and the inset of panel F is 400x. Original magnification for all other photomicrographs is 200x.

The adult and pediatric brain demonstrated diffusely high levels of staining for 5hmC. Similar to the developing fetus, the grey matter of the adult and pediatric cortex showed strong staining in all cell types including neurons, astrocytes, and oligodendroglial cells (Figure 2A and E). The adult and pediatric white matter, while reduced compared to the grey matter, also showed relatively high levels of 5hmC staining (Figure 2B and F). Interestingly, the staining levels within the oligodendroglial population was more variable, with some showing nearly no staining, suggesting epigenetic plasticity in oligodendroglial populations (see inset in Figure 2F). In the cerebellum, both purkinje cells and granule layer neurons showed some degree of staining for 5hmC (Figure 2C and G). The staining levels for 5hmC were also high in non-neuronal elements such as the endothelial cells of the cerebral vasculature (Figure 2D and H). Taken together these results suggest that 5hmC levels are low in the neuronal progenitor cell populations and increase coincident with differentiation and maturation.

### 5hmC Immunohistochemistry in Human Glioma

**Figure 3:**
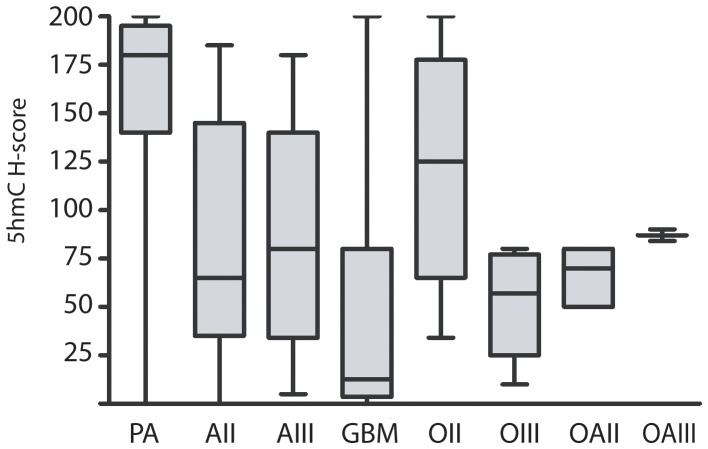
5hmC levels are reduced in glioblastoma. Semi-quantitative intensity scores for 5hmC staining were determined for 225 brain tumors with various histologic diagnoses and grades. Among astrocytic tumors, pilocytic astrocytomas (PA) showed relatively high levels of staining for 5hmC compared to infiltrating astrocytomas. Glioblastoma (GBM) showed significant reduction of 5hmC staining compared to grade II (AII) and grade III astrocytomas (AIII). In oligodendroglial tumors, Grade II oligodendrogliomas (OII) showed high level staining. A reduction in 5hmC levels was observed in grade III oligodendroglioma (OIII). Both grade II and grade III mixed gliomas (OAII and OAIII) showed moderate staining for 5hmC.

**Figure 4:**
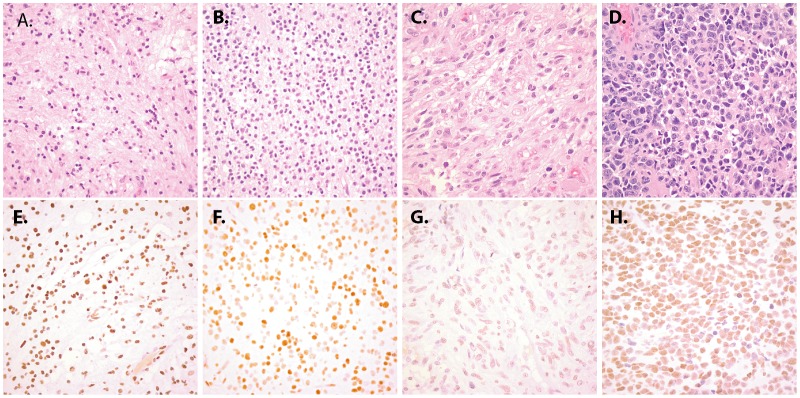
Representative 5hmC staining in glioma. Human brain tumor tissue sections were stained with hematoxylin and eosin (A–D) or with antibodies specific for 5hmC (E–H). Relatively high levels of 5hmC staining was seen in pilocytic astroycytomas (E) and oligodendrogliomas (F). In contrast, glioblastomas overall had reduced 5hmC levels (G). A rare glioblastoma showing relatively high levels of 5hmC staining is shown in panel (H). Original magnification for all photomicrographs is 200x.

We next evaluated 5hmC staining patterns in 225 glial tumors. The patient characteristics of our tumor cohort are presented in Table 1. Interestingly, 5hmC levels were significantly associated with tumor grade. Pilocytic astrocytomas (WHO grade I, n = 66) consistently showed high levels of 5hmC (mean H-score = 164.1, Figure 3 and 4 and Table 2). Compared to pilocytic astroyctomas, infiltrating astroyctomas (WHO grade II-IV) showed a significant reduction of 5hmC staining (p<0.001 for all comparisons). Furthermore, glioblastoma showed a 45 and 48% reduction of 5hmC staining compared to grade II and III astroctyomas (p = 0.006 and p = 0.003, respectively) (See Figure 3 and 4). No difference between 5hmC levels was identified in 35 pediatric compared to 65 adult glioblastomas (p = 0.89).

**Table 1:** Patient cohort characteristics.

		Mean age	Male:Female
Diagnosis	N	(range)	ratio
Pilocytic Astrocytoma	64	12.6 (0.5–51)	1.5
Diffuse Astrocytoma	22	36.7 (15–57)	1.5
Anaplastic Astrocytoma	19	37.5 (10–62)	1.4
Glioblastoma	100	39 (0–86)	1.1
*Adult*	*65*	*53.7 (20–86)*	*1.1*
*Pediatric*	*35*	*11.5 (0–19)*	*1.1*
Oligodendroglioma	11	45.7(28–64)	3.0
Anaplastic Oligodendroglioma	4	39.3 (33–45)	1.0
Oligoastrocytoma	3	39.7 (37–41)	2.0
Malignant Oligoastrocytoma	2	47.5 (28–67)	1.0
Total	225		

**Table 2:** Summary of 5hmC immunohistochemistry.

		Mean	IDH1	H-score		
		5hmC	R132H	IDH1	H-score	
Diagnosis	N	H-score	mutants	R132H	IDH1neg	p-value
Pilocytic Astrocytoma	64	165.1	ND	ND	ND	ND
Diffuse Astrocytoma	22	86.6	10/20	75.0	89.0	0.62
Anaplastic Astrocytoma	19	85.6	13/19	59.8	97.5	0.22
Glioblastoma	100	44.6	9/100	46.4	26.1	0.28
*Adult*	*65*	*44.3*	*4/65*	43.9	51.3	
*Pediatric*	*35*	*45.0*	*5/35*	51.6	6.0	
Oligodendroglioma	12	120.8	11/12	100.0 (a)	122.6	ND
Anaplastic Oligodendroglioma	4	51.0	3/4	80.0 (b)	41.3	ND
Oligoastrocytoma	3	66.7	1/3	65.0	70.0 (c)	ND
Malignant Oligoastrocytoma	2	87.0	0/2	87.0	N/A	ND
Total	225					

For (a) and (b) values are based on a single IDH1 negative tumor. For (c) the value is based on a single IDH1-positive tumor. N = number of tumors of the designated histologic type. IDH R132H mutants = tumors that were immunopositive for antibodies against IDH1 mutant R132H. IDH1neg = tumors immunonegative for IDH1 mutant R132H. ND = not determined due to the absence of IDH1 mutant pilocytic astrocytomas.

Oligodendroglial tumors including oligodendrogliomas (WHO grade II, n = 12), anaplastic oligodendrogliomas (WHO grade III, n = 4), oligoastrocytomas (WHO grade II, n = 2) and malignant oligoastrocytomas (WHO grade III, n = 2) showed overall high levels of 5hmC staining. In fact, among infiltrating gliomas, grade II oligodendrogliomas showed the highest levels of 5hmC (mean H-score = 120.8) (Table 2 and Figure 4B,F). Similar to the pattern seen in astrocytic tumors, anaplastic oligodendroglioma (WHO grade III) showed a trend for reduced H-score compared to grade II oligodendrogliomas (p = 0.052).

### Relationship of 5hmC Immunohistochemistry to IDH1 R132H Mutation Status

**Figure 5:**
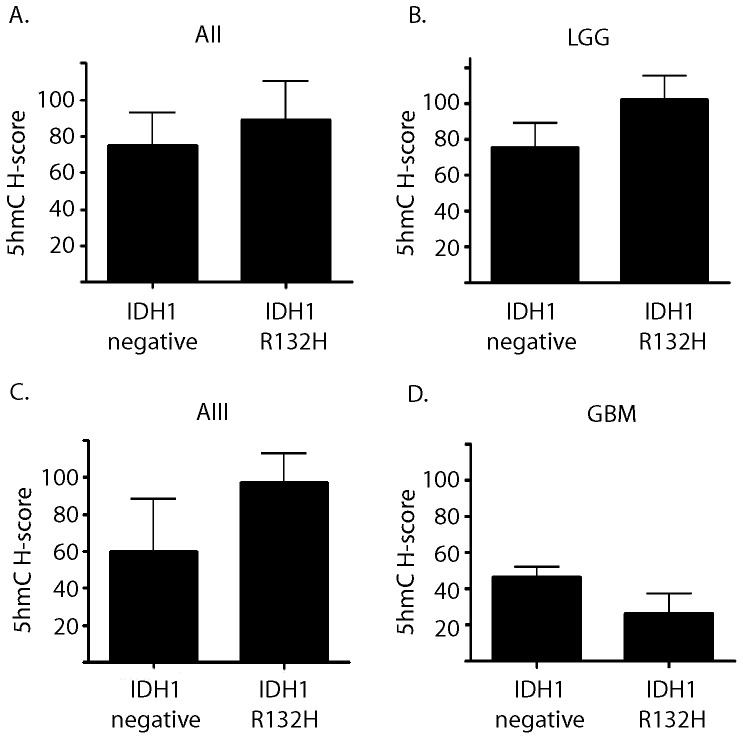
IDH1 R132 H mutation status is not associated with a detectable difference in 5hmC content. Tumors were evaluated for immunoreactivity with antibodies specific for the IDH1 mutant R132H and the corresponding 5hmC levels were correlated with IDH1 immunopositive and immunonegative (IDH1 negative) tumors in (A) grade II astrocytoma, (B) combined low grade gliomas (C) anaplastic astrocytomas, and (D) glioblastoma. No significant difference was identified in 5hmC staining level within any of the specific tumor types.

Given the recent evidence suggesting that mutations in the metabolic enzymes *IDH1* and *IDH2* could be associated with reduced global 5hmC levels [17], [26], we investigated the relationship between *IDH1* mutation status and 5hmC levels. *IDH1* mutation status was determined using an antibody specific to the R132H mutant form of the protein. The R132H alteration is the most frequently detected mutation, accounting for approximately 90% of all IDH changes in brain tumors [19], [33], [34]. The specificity of staining was validated using 10 gliomas with sequence proven *IDH1* R132H and 5 *IDH1* wildtype tumors (data not shown). In each tumor type examined, we failed to detect a relationship between 5hmC staining in *IDH1* R132H-immunoreactive tumors compared to *IDH1* R132H-immunonegative tumors (see Figure 5), including diffuse astrocytoma, anaplastic astrocytomas, and glioblastomas. Only the glioblastoma group showed a mean reduction in H-score in the *IDH1* R132H mutant tumors (46.4 vs 26.1), but the relationship did not reach statistical significance (p = 0.28). We were unable to compare *IDH1* R132H mutant and IDH1-immunonegative tumors in oligodendroglioma because only one tumor in our cohort was negative for the *IDH1* R132H mutation by immunohistochemistry. However, a comparison of combined *IDH1*R132H mutant and IDH1-immunonegative low grade gliomas (grade II astrocytoma, oligodendrogliomas, and mixed oligoastrocytomas) also failed to demonstrate a significant difference (p = 0.21).

### Low 5hmC Staining Level is Associated with Poor Prognosis in Adult Glioblastoma and Anaplastic Astrocytoma

**Figure 6:**
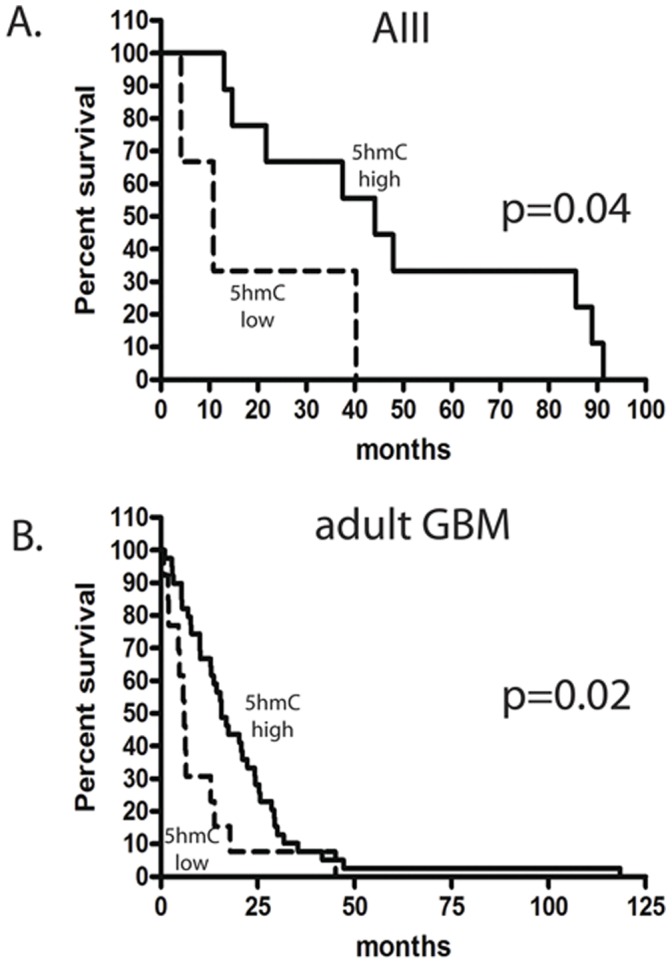
5hmC immunohistochemistry is prognostic of survival in anaplastic astrocytoma and adult glioblastoma. Patients with anaplastic astroyctoma (A) or adults with glioblastoma (B) were stratified with respect to 5hmC staining level into 5hmC-low (first quartile) and 5hmC-high tumors (quartiles 2–4). The percent survival was graphed using the product limit (Kaplan Meier) method. Log rank test of the curves revealed a significant survival advantage in the 5hmC-high group compared to the 5hmC-low group for anaplastic astrocytoma (p = 0.04) and adult glioblastoma (p = 0.02).

The striking association of 5hmC with tumor grade prompted us to evaluate the prognostic significance of 5hmC in the 69 glioblastomas and 21 grade II or III astrocytomas for which survival data was available. We noted that even within grade there was variability such that while many tumors showed very low levels of 5hmC staining, a subset of tumors showed higher staining intensities. We therefore dichotomized tumors within grade into 5hmC-high (H-score >25^th^ percentile) and 5hmC-low (H-score ≤25^th^ percentile) groups, and compared the survival outcomes between these groups. In both pediatric and adult glioblastoma, 5hmC-low specimens were associated with reduced median survival (median survival 6.0 vs. 15.6 months in 5hmC-low compared to 5hmC-high tumors for adult glioblastoma and 13.1 vs. 16.9 months in pediatric glioblastomas) (Table 3). This relationship was also observed in anaplastic astrocytoma (median survival 10.9 months vs. 44.2 months). Comparison of the survival curves revealed significantly reduced survival in the 5hmC-low group for adult glioblastoma (p = 0.02) and anaplastic astrocytoma (p = 0.04) (Figure 6A and B).

**Table 3:** Relationship between 5hmC level and survival in glioma.

				all other	
	5hmC	All Other	5hmC Low	tumors	
	low	tumors	median	median	
Diagnosis	(N)	(N)	survival	survival	P-value
Adult Glioblastoma	13	39	6.0	15.6	0.02 *
Ped. Glioblastoma	6	12	13.1	16.9	0.24
Grade III Astrocytoma	3	9	10.9	44.2	0.04*
Grade II Astrocytoma	3	7	67.3	48.1	0.74

Survival data for glioma patients showing low 5hmC compared to other tumors within grade. Survival values are represented in months. P values were generated using the log rank test. P<0.05 was considered significant. N = number of tumors in the designated group.

To further evaluate the relationship of 5hmC immunohistochemistry and survival we evaluated 5hmC staining in a Cox proportional hazards model including age, gender, 5hmC H-score in the lower 25^th^ percentile, and IDH1 status. In univariate analysis of our glioblastoma cohort, low 5hmC staining score, but not the other variables, was associated with an increased risk of death and shorter survival (p = 0.02; HR = 2.12, 95% CI = 1.11–4.05) (Table S1). In multivariate analysis, low 5hmC staining levels remained an independent predictor for worse prognosis when the model was adjusted for age (p = 0.03; HR = 2.08; CI = 1.08–3.99) or age, sex, gender, and IDH1 status (p = 0.017; HR = 2.28; CI = 1.16–4.49) (Table S2).

Although low 5hmC staining level showed a non-significant trend in the Cox proportional hazards model for anaplastic astrocytoma (p = 0.06, HR = 4.27, 95%CI = 0.94–19.52), none of the variables evaluated showed a significant effect on survival in univariate or multivariate analysis (Table S3). This finding is most likely due to the small number of anaplastic astrocytomas available in our cohort.

### Genes Implicated in 5hmC Homeostasis are Differentially Expressed in Molecular Subtypes of Glioblastoma and Stratify Risk in Glioblastoma

**Figure 7:**
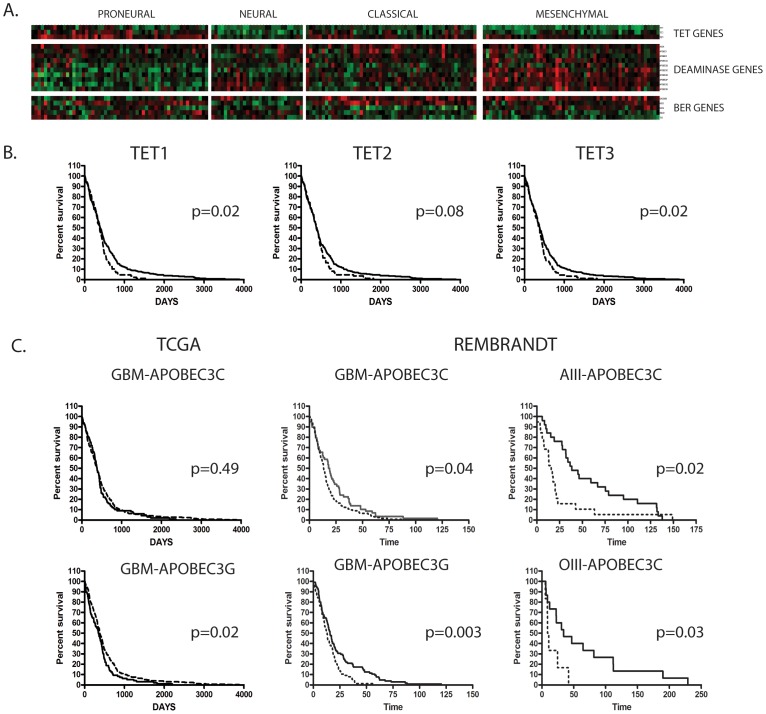
Low expression of TET genes and high expression of APOBEC genes are associated with poor prognosis in malignant glioma. Gene expression signatures were evaluated for the TET genes, AID/APOBEC genes, or genes of the base excision repair pathway (BER) represented in the Verhaak dataset [30]. (A) a heatmap of z-normalized expression values separated into the proneural (P), neural (N), classic (C), and mesenchymal (M) subtypes. Seven of eight genes evaluated were upregulated in the mesenchymal subtype of GBM compared the proneural subtype. Significance of p<0.05, p<0.01, or p<0.001 compared to the proneural subtype are designated by (*), (**), or (***), respectively. Survival analysis was performed for mRNA expression of TET genes (B) and selected APOBEC genes (C) from the TCGA [35] or REMBRANDT [37] datasets. For TCGA data low TET gene expression (dashed line) was designated as the first quartile compared to the remaining three quartiles (solid line). For APOBEC3C and APOBEC3G, high expression (dashed line) was defined as the fourth quartile of expression values compared to the remaining quartiles (solid lines). For REMBRANDT data, a value of ≥2 fold overexpression was used as the cutoff for high expression. P-values <0.05 were considered significant.

Given our observation that low levels of 5hmC were associated with poor prognosis in glioblastoma and anaplastic astroyctoma, we examined whether genes associated with the production or removal of 5hmC tags might also be associated with aggressive behavior in glioma. Using *in silico* analysis, we first evaluated the publically available TCGA dataset [30], [35] to determine if genes previously implicated in 5hmC homeostasis [4], [9]–[12], [36] were associated with specific molecular subtypes of glioblastoma. Included in our analysis were 3 TET enzyme genes (*TET1*, *TET2*, *TET3*), 10 deaminase genes (*AICDA*, *APOBEC1*, *APOBEC2*, *APOBEC3A*, *APOBEC3B*, *APOBEC3C*, *APOBEC3D*, *APOBEC3F*, *APOBEC3G*, *APOBEC3H*), and 5 base excision repair (BER) genes (*TDG*, *SMUG1*, *GADD45B*, *MBD3*, *MBD4*). We found that all three TET enzyme genes showed low expression in the aggressive mesenchymal subtype of glioblastoma, whereas the proneural group showed comparatively higher level expression for each (Figure 7A and Table S4). Evaluation of genes involved in the demethylation pathway revealed 6 of 10 AID/APOBEC genes were increased in the mesenchymal subtype of glioblastoma (Figure 7A and Table S4), whereas 2 of 5 BER genes were increased in the mesenchymal subtype of glioblastoma compared to the proneural subtype (Figure 7A and Table S4).

Next we performed survival analysis on glioblastoma samples from the TCGA to determine whether genes involved in 5hmC homeostasis were associated with reduced survival. We found that low level expression, defined in our cohort as expression values in the first quartile, was associated with reduced survival in *TET1* and *TET3* compared to the remaining tumors. For *TET1*, a reduction of median survival from 393 days to 377.5 days was seen in the low expression group (p = 0.01), whereas in TET3 a reduction in median survival from 382 to 360 days was observed (p = 0.02) (Figure 7B and Table S5). Among the deaminases, we identified one gene, *APOBEC3G*, in which high level expression was associated with reduced survival. A reduction in survival from 383 days to 350 days was seen in the high-expressing group compared to all other tumors (p = 0.02) (Figure 7C and Table S5). A second deaminase *APOBEC3C* showed a reduction in survival between high level expression (quartile one) and low level expression (quartile four) (p = 0.0009).

Univariate analyses of the TCGA dataset revealed a prognostic significance for low expression of *TET1* and *TET3*, or high expression of *APOBEC3G* (p = 0.02 for all genes) (Table S6). *IDH1* mutations (p = 0.001), G-CIMP (p = 0.0001), and age (p = 0.0001) were also associated with reduced survival. (Table S6). On multivariate analysis, low levels of *TET1* and high *APOBEC3G* remained independent predictors for worse prognosis when the model was adjusted for *IDH1* mutation status (p = 0.04, HR = 1.5, 95% CI = 1.03–2.18, and p = 0.03, HR = 1.6, 95% CI = 1.05–2.32, respectively), but did not reach statistical significance when the model was adjusted for age or G-CIMP status (Table S7).

To validate the relationship between high levels of APOBEC genes and reduced survival in an independent dataset, we looked at the overexpression of *APOBEC3G* and *APOBEC3C* in gliomas in the REMBRANDT dataset [37]. Both deaminase genes showed significantly reduced survival in glioblastoma in the high expressing group compared to all other tumors (*APOBEC3C*: p = 0.04, HR = 1.4, CI = 1.02–1.92 and *APOBEC3G*: p = 0.003, HR = 1.6, 1.17–2.18, CI = 1.17–2.18) (Figure 7C and Table S8).In univariate analyses of the REMBRANDT glioblastoma dataset, high APOBEC3C (p = 0.04), high APOBEC3G (p = 0.003), and age (p<0.001) were all associated with reduced survival (Table S9). On multivariate analysis adjusted for age, high *APOBEC3G* remained significantly associated with worse prognosis (p = 0.01, HR = 1.56, CI = 1.1–2.2) (Table S10).

Interestingly, in the REMBRANDT dataset high expression of APOBEC3C was also prognostic in other malignant gliomas including anaplastic astrocytoma and anaplastic oligodendroglioma (p = 0.02 and p = 0.03, respectively) (Figure 7C and Table S3). In a Cox proportional hazards model, high APOBEC3C (p = 0.02) and age (p = 0.004) were significantly associated with worse prognosis in univariate analysis (Table S11). High *APOBEC3C* expression remained significant in anaplastic astrocytoma and showed a trend toward significance in anaplastic oligodendroglioma even when the model was adjusted for age (p = 0.02 and p = 0.07, respectively) (Table S12).

## Discussion

Epigenetic modifications play a crucial role during normal development and adaptive tissue processes, and have been frequently found to be altered in disease states like cancer [1]. Here we explored the distribution of 5hmC in the fetal brain, in the normal pediatric and adult brain, and in neoplastic lesions of the central nervous system. In the developing fetus, more differentiated compartments, including the fetal cortex, showed much higher levels of 5hmC staining compared to the germinal matrix of the forebrain, an area known to contain high levels of neural stem cells and progenitor cells. In adult and pediatric brains, we observed more variable staining among glial cells, but high levels of 5hmC staining in most neuronal populations. Previous studies have identified high levels of 5hmC in neural tissue [6], [7], [13], however because we used immunohistochemistry and included prenatal time points we were able to demonstrate differential staining in progenitor population compared to differentiated tissues. This observation is consistent with data from rodent models demonstrating an overall increase in the 5hmC levels during postnatal brain development [13], and consistent with a recent report showing that tissue-specific progenitor cells outside the brain have low levels of 5hmC [5]. The physiological role of 5hmC in the nervous system is currently unknown, but at least in rodent models, some evidence suggests an interaction of 5hmC with the methyl-binding protein, MeCP2 [13]. MeCP2 has been implicated in neuronal differentiation and maturation [38], [39], and its function is altered in neurodevelopmental diseases like Rett syndrome [40]. A more recent report demonstrated that the methyl-binding domain protein MBD3 specifically binds hydroxymethylated DNA but not methylated DNA and is involved in regulating pluripotency in embryonic stem cells [41]. The binding proteins and signaling pathways associated with changes in 5hmC during neuronal differentiation and brain tumorigenesis warrant more detailed mechanistic investigation.

Epigenetic changes in solid tumors have been well characterized [42]; however, the role of 5hmC in neoplastic diseases is largely unexplored. Recent results have established that 5hmC levels are reduced in neoplastic lesions compared to normal tissue [5], [42]–[44]. In our panel of human brain tumors, we found significant variability between both histologic tumor type and grades. Pilocytic astrocytomas and oligodendrogliomas showed particularly high levels of 5hmC in our cohort. Among infiltrating astrocytomas, 5hmC was associated with tumor grade, with glioblastoma showing lower 5hmC staining levels compared to both grade II and grade III astrocytoma. This finding is similar to that observed by Jin *et al*. in examination of 5hmC levels in 35 astrocytomas by mass spectrometry [16]. Additionally, while the current manuscript was in preparation, another group reported a similar relationship between reduced 5hmC levels and increased tumor grade in 103 gliomas [43]. The current study is distinguished from this recent work by Kraus *et al.* [43] in that we evaluated an expanded glioma dataset with more tumor types, and identified an association between low 5hmC and reduced survival in both glioblastoma and anaplastic astrocytoma. The association of low 5hmC with aggressive behavior in gliomas is supported by previous work demonstrating an inverse relationship between 5hmC levels and Ki67 staining [16].

Differences in 5hmC can be either attributed to (a) decreased generation of 5hmC, or (b) to an increase in removal of the 5hmC mark. Activity of the TET enzymes, which oxidize 5mC to 5hmC, is reportedly inhibited by 2-HG, a product of mutant IDH1/2 enzymes [17], [25]. Recently, Jin et al. failed to detect a relationship between *IDH1* mutation status and 5hmC in astrocytoma [16]. Similarly, we did not detect a difference in 5hmC levels between tumors that were immunoreactive or immunonegative for the *IDH1* mutant R132H within multiple brain tumor types. In fact, oligodendroglioma, a tumor type which were nearly all mutant for IDH1 in our cohort, showed the highest level of staining for 5hmC among infiltrating gliomas. Our results do not rule out the possibility that *IDH1/2* mutant-specific oncometabolites can influence the formation of 5hmC. In fact, recent reports strongly support a role for *IDH1* mutations to both reduce the TET2-dependent oxidation of 5mC to 5hmC and cause G-CIMP in immortalized human astrocytes [45]. However, our data suggests that other factors may also contribute to 5hmC homeostasis. In support of this, reduction in TET2 enzyme levels by promoter methylation has recently been reported in low grade diffuse gliomas lacking *IDH1/2* mutations [46], and loss of the *TET2* locus (4q24) has been reported in approximately 2% of glioblastomas [35]. 5hmC levels could also be reduced through active removal of the mark. Recent evidence suggests that 5hmC is removed through a process that involves initial deamination by activation induced deaminase (AID) or one of the apolipoprotein B mRNA editing enzyme complex (APOBEC) family of deaminases, with subsequent removal of the deaminated base by base excision repair [4], [12], [47]. Additionally, because our IDH1 antibody was specific to only R132H mutation, we could have underestimated the tumors with *IDH1* or *IDH2* mutations. However, the *IDH1* R132H mutations is by far the most prevalent IDH1/2 mutation found in brain tumors, making up 89% of all mutations [34]. Therefore, it is unlikely that a large proportion of IDH1 immuno-negative tumors in our cohort were also IDH1/2 mutant.

Our immunohistochemical data suggests that low levels of 5hmC are strongly associated with reduced survival in malignant glioma. In glioblastoma, low 5hmC is associated with reduced survival even after controlling for IDH1 mutation status, age at diagnosis, and gender. *In silico* analysis of genes that regulate 5hmC homeostasis supports this observation, with mRNA levels of the TET enzymes upregulated in the less aggressive proneural subgroup compared to the mesenchymal subgroup. Low levels of *TET1* and *TET3* were also associated with reduced survival in glioblastoma. The converse is true of the APOBEC genes, which show high level expression in the mesenchymal subgroup, and have two members, *APOBEC3C* and *APOBEC3G* which are associated with reduced survival when expressed at high levels. These findings suggest that decreased formation of 5hmC, potentially due to reduced TET enzyme levels, and/or depletion of 5hmC, potentially through a mechanism involving deamination and base-excision repair, are linked with aggressive behavior in glioma.

There is evidence that the balance between 5mC and 5hmC can regulate stem cell phenotype and differentiation. For instance, knockdown of the TET1 enzyme (and accompanying reductions in 5hmC levels) in embryonic stem cells has been shown previously to regulate markers of pluripotency in a 5mC-dependent manner [9], [48]. It is possible that depletion of 5hmC due to subsequent demethylation promotes a “stem-like” state in tumors. For instance, Guo *et al*. showed that activity of the demethylation pathway could regulate gene expression *in vivo* [12]. While no evaluation of the gene loci regulated by 5hmC and demethylation has been reported for tumors, there is precedent for the regulation of stem cell markers in neural stem cells by DNA methylation. In fact, the neural stem cell marker CD133 has been shown to be regulated by DNA methylation [49], and the mark has shown prognostic significance in some studies of glioma [49]–[51]. While our method of detection of 5hmC only allows for evaluation of global 5hmC levels rather than the distribution of 5hmC at the level of individual genes, a recently described method of oxidatiive bisulfite sequencing [52] could help elucidate the relationship and distribution of 5mC and 5hmC in gene regulation in glioma.

The molecular characteristics of the tumors in our cohort showing the highest level of staining for 5hmC is currently unclear. One possibility is that these represent tumors with the G-CIMP phenotype. The G-CIMP subgroup is enriched in the proneural transcriptional class and has a high proportion of *IDH1/2* mutations [22], [45]. Evaluation of the TCGA dataset with respect to mRNA expression of TET enzymes and deaminase genes shows that the G-CIMP tumors and *IDH1* mutant tumors both exhibit significantly higher expression of TET genes and low levels of deaminase genes compared to non-CIMP tumors or *IDH1* wild type tumors (supplemental Figures S2 and S3). In our cohort only one glioblastoma in the highest quartile of staining was positive for the *IDH1* R132H mutation. While it has been reported that IDH1 mutations are sufficient to produce the G-CIMP phenotype [45], it is currently unclear whether the mutations are necessary. Therefore, it is possible that the tumors showing the very highest level of staining for 5hmC represent an *IDH1* wildtype, G-CIMP group. Most commonly utilized methods to detect DNA methylation, including bisulfite sequencing, are unable to differentiate 5mC and 5hmC and therefore heterogeneity between the two bases would not be appreciated. Further studies are warranted to evaluate the possible link between 5hmC and IDH mutations in G-CIMP positive tumors.

In summary, we identified low 5hmC levels in association with progenitor regions in the brain, but elevated 5hmC levels in more differentiated regions in the fetal, pediatric, and adult brains. Among human brain neoplasms, low grade tumors showed high 5hmC levels, whereas within glial lineages, high grade tumors showed reduced 5hmC staining. Our findings suggest that 5hmC may be intricately involved in differentiation in the nervous system both in normal and neoplastic conditions. Importantly, we found low 5hmC was associated with reduced survival in adult glioblastoma and anaplastic astrocytoma, suggesting that the mechanisms responsible for regulating 5hmC may represent a potential future therapeutic target.

## Supporting Information

Figure S1 **TET2 expression shows the greatest increase during murine neurogenesis.**
(PDF)

Figure S2 **IDH1 mutant tumors show differential expression of genes in the demethylase pathway compared to IDH1 wildtype tumors.**
(PDF)

Figure S3 **G-CIMP tumors show differential expression of genes in the demethylase pathway compared to non-G-CIMP tumors.**
(PDF)

Table S1 **Univariate Cox proportional hazards analysis for glioblastoma tissue microarray.**
(PDF)

Table S2 **Multivariate Cox proportional hazards analysis for glioblastoma tissue microarray.**
(PDF)

Table S3 **Univariate Cox proportional hazards analysis for anaplastic astrocytoma tissue microarray.**
(PDF)

Table S4 **Expression of genes involved in 5hmC homeostasis are associated with specific transcriptional class of glioblastoma.**
(PDF)

Table S5 **Survival analysis from TCGA dataset for TET or selected APOBEC genes in glioblastoma.**
(PDF)

Table S6 **Univariate Cox proportional hazards model for TCGA glioblastoma dataset.**
(PDF)

Table S7 **Multivariate Cox proportional hazards model for TCGA glioblastoma dataset.**
(PDF)

Table S8 **Survival analysis from REMBRANDT database for selected APOBEC genes in gliomas.**
(PDF)

Table S9 **Univariate Cox proportional hazards analysis for glioblastoma from the REMBRANDT dataset.**
(PDF)

Table S10 **Multivariate Cox proportional hazards analysis of glioblastoma from the REMBRANDT dataset.**
(PDF)

Table S11 **Univariate Cox proportional hazards analysis for anaplastic astrocytoma in the REMBRANDT dataset.**
(PDF)

Table S12 **Multivariate Cox proportional hazards analysis for anaplastic astrocytoma in the REMBRANDT dataset.**
(PDF)
