# Supplementary material for: Decreased 5-Hydroxymethylcytosine Is Associated with Neural Progenitor Phenotype in Normal Brain and Shorter Survival in Malignant Glioma
Source: PLoS One. 2012 Jul 19;7(7):e41036. doi: 10.1371/journal.pone.0041036 (PMC3400598; doi:10.1371/journal.pone.0041036)
Supplement: Figure S1 — TET2 expression shows the greatest increase during murine neurogenesis. (PDF) [file pone.0041036.s001.pdf]

Figure S1.

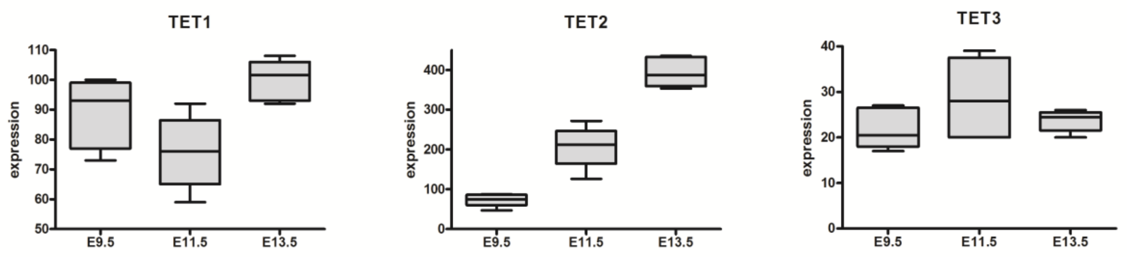

Figure S1. TET2 expression shows the greatest increase during murine neurogenesis. Normalized mRNA expression values for TET isoforms were downloaded from GEOdatasets record GDS3442. Expression values correspond to Affymetrix arrays performed on brains of C57Bl/6 mice at E9.5, E11.5, and E13.5. These time points correspond to a period before neurogenesis when predominantly neural precursor cells are present (E9.5), a period during neurogenesis (E11.5), and the peak of neurogenesis (E13.5). TET2 showed the greatest increase during neurogenesis compared to the other TET isoforms.
