# Supplementary material for: Decreased 5-Hydroxymethylcytosine Is Associated with Neural Progenitor Phenotype in Normal Brain and Shorter Survival in Malignant Glioma
Source: PLoS One. 2012 Jul 19;7(7):e41036. doi: 10.1371/journal.pone.0041036 (PMC3400598; doi:10.1371/journal.pone.0041036)
Supplement: Figure S2 — IDH1 mutant tumors show differential expression of genes in the demethylase pathway compared to IDH1 wildtype tumors. (PDF) [file pone.0041036.s002.pdf]

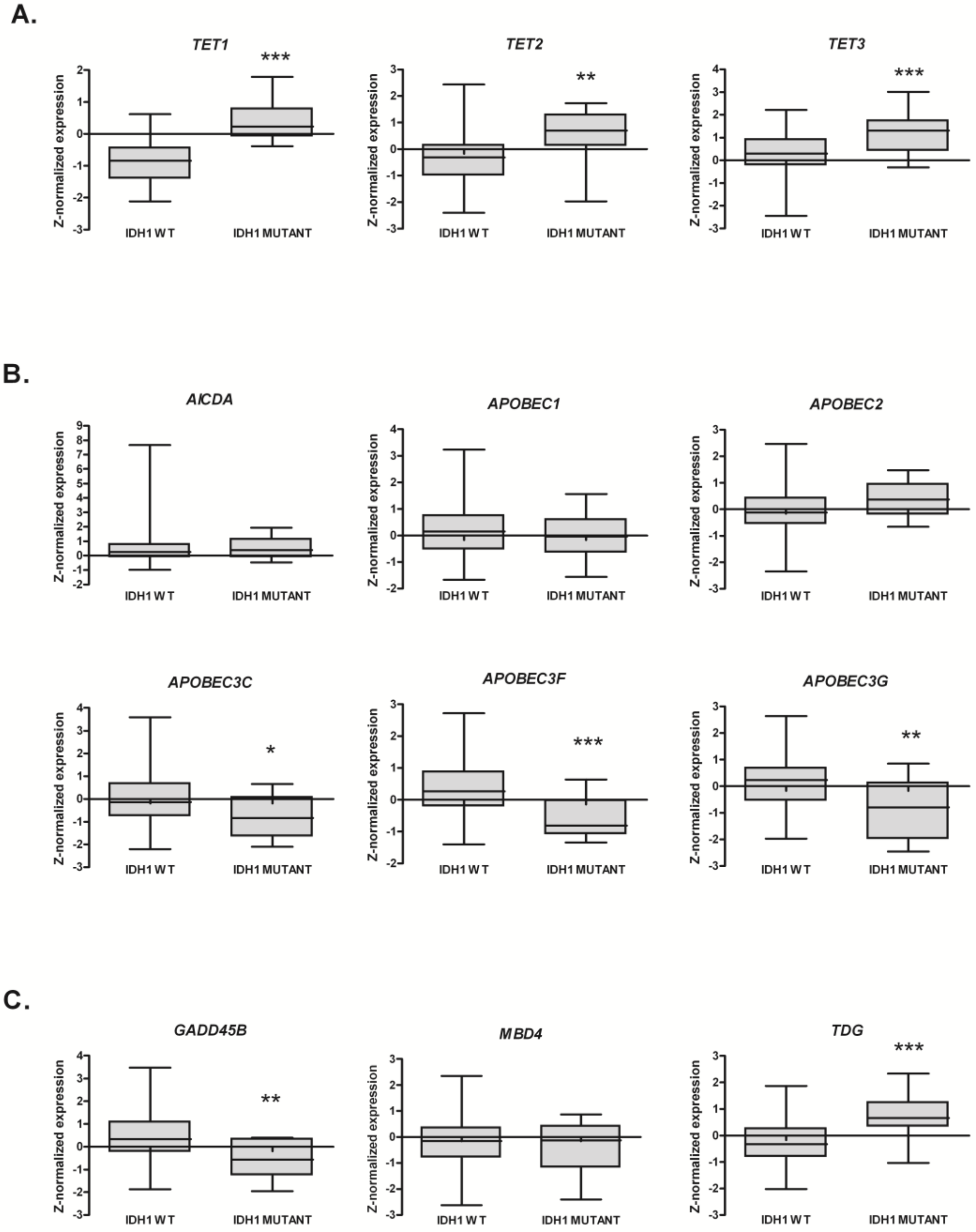

Figure S2. IDH1 mutant tumors show differential expression of genes in the demethylase pathway compared to IDH1 wildtype tumors. Z-normalized gene expression values for TET enzymes (a), deaminase genes (b), or base excisioin repair genes (c) implicated in the active demethylase pathway were evaluated in IDH1 mutant versus IDH1 wildtype tumors from the TCGA dataset [REF]. Differences in expression were determined using the Student's t-test. P-value ≤ 0.05 were considered statistically significant. (*) = p<0.05, (**)= p<0.001, (***)= p<0.0001.
