# Supplementary material for: Decreased 5-Hydroxymethylcytosine Is Associated with Neural Progenitor Phenotype in Normal Brain and Shorter Survival in Malignant Glioma
Source: PLoS One. 2012 Jul 19;7(7):e41036. doi: 10.1371/journal.pone.0041036 (PMC3400598; doi:10.1371/journal.pone.0041036)
Supplement: Figure S3 — G-CIMP tumors show differential expression of genes in the demethylase pathway compared to non-G-CIMP tumors. (PDF) [file pone.0041036.s003.pdf]

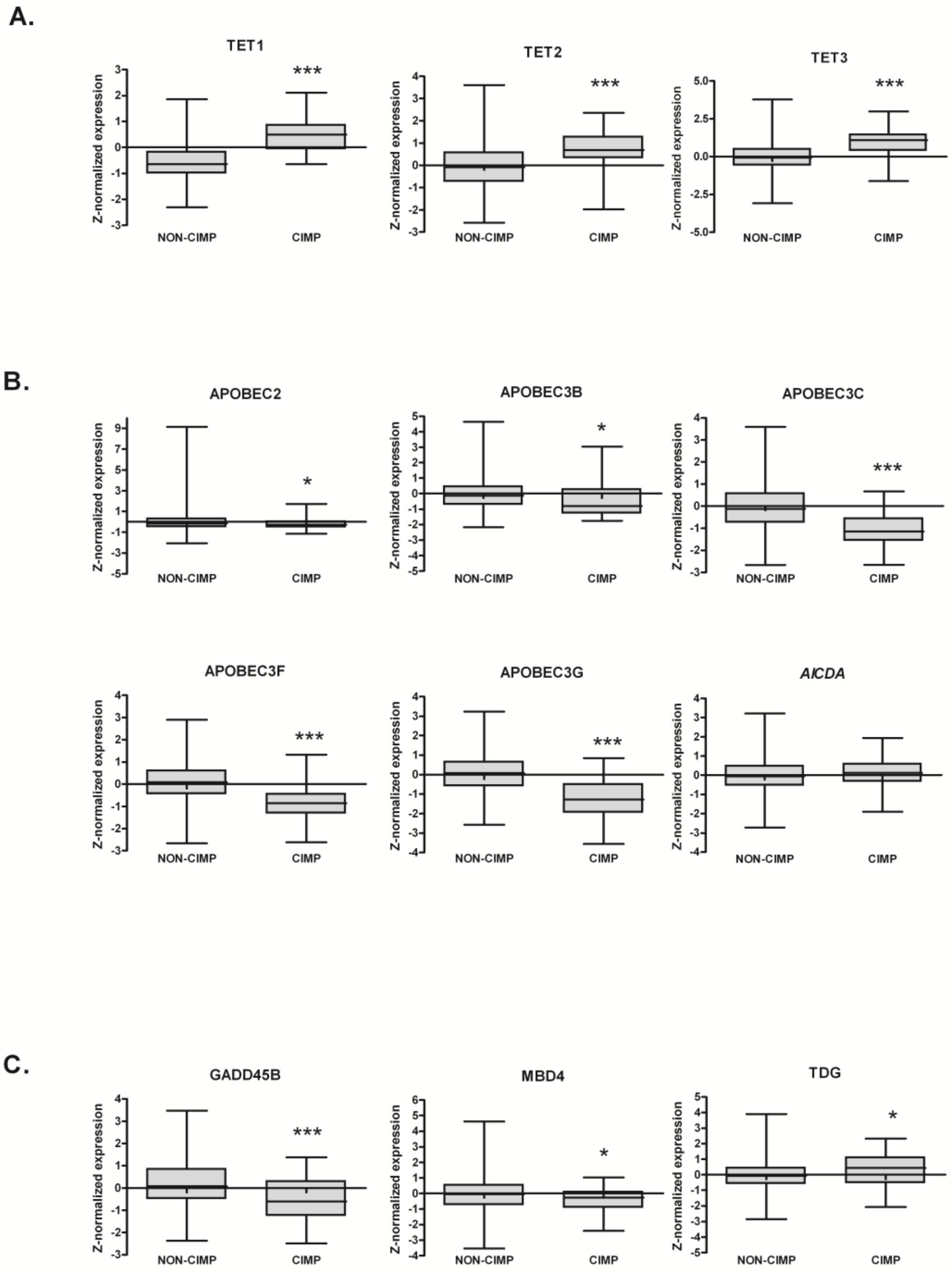

Figure S3. G-CIMP tumors show differential expression of genes in the demethylase pathway compared to non-G-CIMP tumors. Z-normalized gene expression values for TET enzymes (a), deaminase genes (b), or base excisioin repair genes (c) implicated in the active demethylase pathway were evaluated in G-CIMP versus non-G-CIMP tumors from the TCGA dataset [REF]. Differences in expression were determined using the Student's t-test. P-value ≤ 0.05 were considered statistically significant. (*) = p<0.05, (**)= p<0.001, (***)= p<0.0001.
