# Supplementary material for: Decreased 5-Hydroxymethylcytosine Is Associated with Neural Progenitor Phenotype in Normal Brain and Shorter Survival in Malignant Glioma
Source: PLoS One. 2012 Jul 19;7(7):e41036. doi: 10.1371/journal.pone.0041036 (PMC3400598; doi:10.1371/journal.pone.0041036)
Supplement: Table S2 — Multivariate Cox proportional hazards analysis for glioblastoma tissue microarray. (PDF) [file pone.0041036.s005.pdf]

**Table S2. Multivariate Cox proportional hazards analysis for glioblastoma tissue microarray**

| Variable | Reference | HR | CI(95%) | p-value |
|---|---|---|---|---|
| Low 5hmC[a] | High 5hmC | 2.08 | 1.08-3.99 | 0.03 |
| Low 5hmC[b] | High 5hmC | 2.28 | 1.16-4.49 | 0.02 |

Low 5hmC= H-score in the first quartile; High 5hmC= H-score in the top three quartiles. The hazard ratio (HR) for all reference variables was set to 1. P-value < 0.05 was considered statistically significant.
*a* = multivariate Cox model adjusted for age
*b* = multivariate analysis adjusted for age, gender, and IDH1 R132H mutation status
