# Supplementary material for: Decreased 5-Hydroxymethylcytosine Is Associated with Neural Progenitor Phenotype in Normal Brain and Shorter Survival in Malignant Glioma
Source: PLoS One. 2012 Jul 19;7(7):e41036. doi: 10.1371/journal.pone.0041036 (PMC3400598; doi:10.1371/journal.pone.0041036)
Supplement: Table S3 — Univariate Cox proportional hazards analysis for anaplastic astrocytoma tissue microarray. (PDF) [file pone.0041036.s006.pdf]

Table S3. Univariate Cox proportional hazards analysis for anaplastic astrocytoma tissue microarray

Variable	Reference	HR	CI(95%)	p-value
Low 5hmC	High 5hmC	4.27	0.94-19.52	0.06
IDH1 R132H (+)	IDH1 R132H (-)	0.72	0.15-3.49	0.69
Gender (male)	Gender (female)	0.78	0.23-2.58	0.68
Age	*	1.08	0.99-1.18	0.09

For the categorical variables: Low 5hmC= H-score the first quartile; High 5hmC= H-score in the top three quartiles; IDH1 R132H mutation status was assessed by immunohistochemistry. Age was evaluated as a continuous variable. The hazard ratio (HR) for all reference variables was set to 1. P-value < 0.05 was considered statistically significant.
