# Supplementary material for: Decreased 5-Hydroxymethylcytosine Is Associated with Neural Progenitor Phenotype in Normal Brain and Shorter Survival in Malignant Glioma
Source: PLoS One. 2012 Jul 19;7(7):e41036. doi: 10.1371/journal.pone.0041036 (PMC3400598; doi:10.1371/journal.pone.0041036)
Supplement: Table S4 — Expression of genes involved in 5hmC homeostasis are associated with specific transcriptional class of glioblastoma. (PDF) [file pone.0041036.s007.pdf]

Table S4. Expression of genes involved in 5hmC homeostasis are associated with specific transcriptional class of glioblastoma

Gene	Mean Proneural Expression	Mean Mesenchymal Expression	p-value
TET1	-0.2871 ± 0.1115	-1.1660 ± 0.0665	<0.0001
TET2	0.2795 ± 0.1117	-0.5869 ± 0.0980	<0.0001
TET3	1.0310 ± 0.1058	0.2393 ± 0.0711	<0.0001
APOBEC1	-0.0120 ± 0.0959	-0.2528 ± 0.1537	0.003
APOBEC2	0.1129 ± 0.0797	-0.0897 ± 0.1133	0.1465
APOBEC3A	-0.0773 ± 0.0829	0.4315 ± 0.1177	0.0006
APOBEC3B	-0.2688 ± 0.1250	0.0669 ± 0.1238	0.0591
APOBEC3C	-0.6832 ± 0.1226	0.7499 ± 0.1229	<0.0001
APOBEC3D	-0.5891 ± 0.1423	0.6852 ± 0.1277	<0.0001
APOBEC3F	-0.4428 ± 0.0810	0.7478 ± 0.0864	<0.0001
APOBEC3G	-0.7525 ± 0.1204	0.5259 ± 0.0915	<0.0001
APOBEC3H	-0.4049 ± 0.1155	0.6838 ± 0.1442	<0.0001
AICDA	0.4020 ±0.1494	0.3231 ± 0.0922	0.65
TDG	0.1231 ± 0.1125	-0.5774 ± 0.0898	<0.0001
SMUG1	0.3973 ± 0.2045	-0.2719 ± 0.1090	0.0047
MBD3	0.5511 ± 0.1383	0.2527 ± 0.0930	0.0762
MBD4	-0.3731 ± 0.1079	0.2727 ± 0.1124	<0.0001
GADD45B	-0.3136 ± 0.1076	0.8881 ± 0.1259	<0.0001

Expression values in the TCGA Proneural and Mesenchymal transcriptional classes were evaluated using Studen'ts t-test. P values ≤ 0.05 were considered significant.
