# Supplementary material for: Decreased 5-Hydroxymethylcytosine Is Associated with Neural Progenitor Phenotype in Normal Brain and Shorter Survival in Malignant Glioma
Source: PLoS One. 2012 Jul 19;7(7):e41036. doi: 10.1371/journal.pone.0041036 (PMC3400598; doi:10.1371/journal.pone.0041036)
Supplement: Table S5 — Survival analysis from TCGA dataset for TET or selected APOBEC genes in glioblastoma. (PDF) [file pone.0041036.s008.pdf]

Table S5. Survival analysis from TCGA dataset for TET or selected APOBEC genes in glioblastoma

GENE	Low (N)	High (N)	Low median survival (days)	High median survival (days)	P-value
TET1	88	261	377.5	393	0.02*
TET2	86	263	396	384	0.08
TET3	91	296	360	382	0.02*
APOBEC3C	287	100	377	382	0.48
APOBEC3G	292	96	383	350	0.02*

Survival data for glioma patients were downloaded from the TCGA dataset [31] and separated into low and high expression groups. Survival values are represented in days. For TET genes low expression was designated the lowest quartile of expression values, whereas high expression refers to all other tumors. For APOBEC genes, high expression refers to the highest quartile of expression values, whereas low expression refers to the bottom three quartiles. P values were generated using the log rank test. P<0.05 was considered significant.
