# Supplementary material for: Decreased 5-Hydroxymethylcytosine Is Associated with Neural Progenitor Phenotype in Normal Brain and Shorter Survival in Malignant Glioma
Source: PLoS One. 2012 Jul 19;7(7):e41036. doi: 10.1371/journal.pone.0041036 (PMC3400598; doi:10.1371/journal.pone.0041036)
Supplement: Table S6 — Univariate Cox proportional hazards model for TCGA glioblastoma dataset. (PDF) [file pone.0041036.s009.pdf]

Table S6. Univariate Cox proportional hazards model for TCGA glioblastoma dataset

Variable	Reference	HR	CI(95%)	p-value
Low TET1	High TET1	1.33	1.04-1.71	0.02
Low TET3	High TET3	1.32	1.04-1.68	0.02
High APOBEC3G	Low APOBEC3G	1.31	1.04-1.65	0.02
IDH1 Mutation	WT IDH1	0.29	0.14-0.58	0.001
G-CIMP	Non-G-CIMP	0.35	0.23-0.52	0.0001
Gender (male)	Gender(female)	1.15	0.92-1.43	0.21
Age	*	1.03	1.03-1.04	0.0001

For the categorical variables Low TET1 and TET3= z-normalized mRNA expression in the first quartile; high TET1 and TET3= z-normalized mRNA expression in the highest three quartiles; High APOBEC3G= z-normalized mRNA expression in the fourth quartile; Low APOBEC3G= z-normalized mRNA expression in the lowest three quartiles; G-CIMP=glioma-CpG island methylator phenotype; Age was evaluated as a continuous variable. The hazard ratio (HR) for all reference variables was set to 1. P-value <0.05 was considered statistically significant.
