# Supplementary material for: Decreased 5-Hydroxymethylcytosine Is Associated with Neural Progenitor Phenotype in Normal Brain and Shorter Survival in Malignant Glioma
Source: PLoS One. 2012 Jul 19;7(7):e41036. doi: 10.1371/journal.pone.0041036 (PMC3400598; doi:10.1371/journal.pone.0041036)
Supplement: Table S7 — Multivariate Cox proportional hazards model for TCGA glioblastoma dataset. (PDF) [file pone.0041036.s010.pdf]

Table S7. Multivariate Cox proportional hazards model for TCGA glioblastoma dataset

Variable	Reference	HR	CI(95%)	p-value
Low TET1[a]	High TET1	1.50	1.03-2.18	0.04
Low TET1[b]	High TET1	1.27	0.87-1.87	0.22
Low TET2[a]	High TET3	1.48	0.82-2.56	0.19
Low TET3[b]	High TET3	1.29	0.72-2.33	0.39
High APOBEC3G[a]	Low APOBEC3G	1.56	1.04-1.65	0.02
High APOBEC3G[b]	Low APOBEC3G	1.37	0.91-2.06	0.13

For the categorical variables Low TET1 and TET3= z-normalized mRNA expression in the first quartile; high TET1 and TET3= z-normalized mRNA expression in the highest three quartiles; High APOBEC3G= z-normalized mRNA expression in the fourth quartile; Low APOBEC3G= z-normalized mRNA expression in the lowest three quartiles. The hazard ratio (HR) for all reference variables was set to 1. P-value <0.05 was considered statistically significant. a = adjusted for IDH1 mutation status; b = adjusted for age, IDH1 mutation status, and G-CIMP phenotype.
