# Supplementary material for: Decreased 5-Hydroxymethylcytosine Is Associated with Neural Progenitor Phenotype in Normal Brain and Shorter Survival in Malignant Glioma
Source: PLoS One. 2012 Jul 19;7(7):e41036. doi: 10.1371/journal.pone.0041036 (PMC3400598; doi:10.1371/journal.pone.0041036)
Supplement: Table S8 — Survival analysis from REMBRANDT database for selected APOBEC genes in gliomas. (PDF) [file pone.0041036.s011.pdf]

Table S8. Survival analysis from REMBRANDT database for selected APOBEC genes in glioma

APOBEC3C					
**Diagnosis**	**Low (N)**	**High (N)**	**Low median survival (months)**	**High median survival (months)**	**P-value**
Grade II astrocytoma	31	19	67.8	29.5	0.15
Grade III astrocytoma	25	19	38.4	15.8	0.02*
Glioblastoma	58	123	18.0	13.4	0.04*
Grade II oligo	14	5	37.8	19.6	0.64
Grade III oligo	15	6	34.1	9.7	0.03*
**APOBEC3G**					
**Diagnosis**	**Low (N)**	**High (N)**	**Low median survival**	**High median survival**	**P-value**
Grade II astrocytoma	39	11	66.4	15.0	0.02*
Grade III astrocytoma	27	17	31.7	17.7	0.29
Glioblastoma	105	76	15.8	12.6	0.003*
Grade II oligo	17	2	36.8	33.2	0.94
Grade III oligo	18	3	27.6	8.8	0.23

Survival data for glioma patients showing low or high APOBEC gene expression were downloaded from the REMBRANDT database [37]. High expression refers to the highest quartile of expression values, whereas low expression refers to the bottom three quartiles. P values were generated using the log rank test. P<0.05 was considered significant.
