# Supplementary material for: Decreased 5-Hydroxymethylcytosine Is Associated with Neural Progenitor Phenotype in Normal Brain and Shorter Survival in Malignant Glioma
Source: PLoS One. 2012 Jul 19;7(7):e41036. doi: 10.1371/journal.pone.0041036 (PMC3400598; doi:10.1371/journal.pone.0041036)
Supplement: Table S9 — Univariate Cox proportional hazards analysis for glioblastoma from the REMBRANDT dataset. (PDF) [file pone.0041036.s012.pdf]

Table S9. Univariate Cox proportional hazards analysis for glioblastoma from the REMBRANDT dataset

Variable	Reference	HR	CI(95%)	p-value
High APOBEC3C	Low APOBEC3C	1.4	1.02-1.92	0.04
High APOBEC3G	Low APOBEC3G	1.6	1.17-2.18	0.003
Gender (male)	Gender (female)	1.21	0.84-1.75	0.30
Age	*	1.24	1.16-1.34	<0.001

For the categorical variables High APOBEC3C= mRNA expression ≥ 2-fold above mean; Low APOBEC3C= mRNA expression <2-fold above mean; High APOBEC3G= mRNA expression ≥ 2-fold above mean; Low APOBEC3G= mRNA expression <2-fold above mean; Age was evaluated as a continuous variable. The hazard ratio (HR) for all reference variables was set to 1. P-value < 0.05 was considered statistically significant.
