# Supplementary material for: Decreased 5-Hydroxymethylcytosine Is Associated with Neural Progenitor Phenotype in Normal Brain and Shorter Survival in Malignant Glioma
Source: PLoS One. 2012 Jul 19;7(7):e41036. doi: 10.1371/journal.pone.0041036 (PMC3400598; doi:10.1371/journal.pone.0041036)
Supplement: Table S11 — Univariate Cox proportional hazards analysis for anaplastic astrocytoma in the REMBRANDT dataset. (PDF) [file pone.0041036.s014.pdf]

Table S11. Univariate Cox proportional hazard analysis for anaplastic astrocytoma in the REMBRANDT dataset

Variable	Reference	HR	CI(95%)	p-value
High APOBEC3C	Low APOBEC3C	2.14	1.14-4.02	0.02
Gender (male)	Gender (female)	0.55	0.26-1.18	0.13
Age	*	1.20	1.06-1.37	0.004

For the categorical variables High APOBEC3C= mRNA expression ≥ 2-fold above mean; Low APOBEC3C= mRNA expression <2-fold above mean; Age was evaluated as a continuous variable. The hazard ratio (HR) for all reference variables was set to 1. P-value < 0.05 was considered statistically significant.
