# Supplementary material for: Decreased 5-Hydroxymethylcytosine Is Associated with Neural Progenitor Phenotype in Normal Brain and Shorter Survival in Malignant Glioma
Source: PLoS One. 2012 Jul 19;7(7):e41036. doi: 10.1371/journal.pone.0041036 (PMC3400598; doi:10.1371/journal.pone.0041036)
Supplement: Table S12 — Multivariate Cox proportional hazards analysis for anaplastic astrocytoma in the REMBRANDT dataset. (PDF) [file pone.0041036.s015.pdf]

Table S12. Multivariate Cox proportional hazard analysis for anaplastic astrocytoma in the REMBRANDT dataset

Variable	Reference	HR	CI(95%)	p-value
High APOBEC3C[a]	Low APOBEC3C	2.14	1.14-4.02	0.02

High APOBEC3C= mRNA expression ≥ 2-fold above mean; Low APOBEC3C= mRNA expression <2-fold above mean. The hazard ratio (HR) for all reference variables was set to 1. P-value <0.05 was considered statistically significant. a = adjusted for age.
